# Hippocampal Lateralization and Synaptic Plasticity in the Intact Rat: No Left–Right Asymmetry in Electrically Induced CA3-CA1 Long-Term Potentiation

**DOI:** 10.1016/j.neuroscience.2018.11.044

**Published:** 2019-01-15

**Authors:** Stephen J. Martin, Kate L. Shires, Bruno M. da Silva

**Affiliations:** a Division of Systems Medicine, University of Dundee, Ninewells Hospital and Medical School, Dundee DD1 9SY, UK; b Cardiff University Biobank, Cardiff University, Dental Drive, Heath Park, Cardiff CF14 4AX, UK; c Centre for Cognitive and Neural Systems (CCNS), University of Edinburgh, 1 George Square, Edinburgh EH8 9JZ, UK

**Keywords:** LFP, local field potential, LTD, long-term depression, LTP, long-term potentiation, PPF, paired-pulse facilitation, PTP, post-tetanic potentiation, hippocampus, LTP, synaptic plasticity, Schaffer collateral, commissural, *in vivo*

## Abstract

The hippocampus is not a unitary, homogeneous brain area. Anatomical and functional specialization is evident along the septotemporal axis of the structure, and between the left and right hemispheres. In the mouse brain, a left–right asymmetry has been discovered in the plasticity of CA3-CA1 projections originating in the left versus right hippocampus. Presynaptic afferents originating in the left hemisphere—including both uncrossed Schaffer collaterals, and crossed commissural projections to the contralateral CA1—form small, plastic synapses, whereas afferents originating in right CA3 contact larger, less plastic, synapses. Studies using optogenetic techniques to selectively activate fibers originating from one hemisphere in *ex vivo* slices have revealed that projections originating from left CA3 exhibit a far greater capacity for long-term potentiation (LTP) of synaptic strength than those originating on the right. However, corresponding data from rats are currently unavailable, leaving open the question of species differences in hippocampal symmetry. In the current study, we reanalyzed data from our previous *in vivo* LTP work to address this issue. We analyzed plasticity in independent Schaffer collateral and commissural projections to CA1 originating from left and right CA3 in male Lister-hooded rats. However, we found no differences in the magnitude and duration of LTP induced in either crossed or uncrossed pathways following high-frequency tetanization of left versus right CA3. This contrast with previous findings may stem from methodological differences between *in vivo* electrical and *ex vivo* optogenetic approaches, but may reflect a genuine species difference in the organization and laterality of the rodent CA3-CA1 system.

## Highlights

• We analyzed plasticity in inputs to CA1 originating from left and right CA3 in rats.
• There was no left–right asymmetry in long-term potentiation of CA1 synaptic strength.
• Past work has revealed lateralized LTP using an *ex vivo* optogenetic approach in mice.
• There may be a species difference in the organization of the rodent CA3-CA1 system.

## Introduction

The hippocampus is a structurally and operationally heterogeneous structure. There is abundant evidence for functional differentiation along the septotemporal (dorsoventral) axis (de Hoz and Martin, 2014, Strange et al., 2014, Bast, 2011), and functional imaging and neuropsychological studies have long pointed to a division of labor between the left and right hippocampus in humans, with greater right hippocampal involvement in allocentric spatial memory, and a more pronounced role for the left hippocampus in autobiographical or episodic memory (see Burgess et al., 2002). The evidence for lateralization of function in rodents is less clear-cut, but there have been several reports of the effects of unilateral hippocampal lesions in rats. Their interpretation is, however, complicated by the existence of inter-hippocampal commissural projections, as discussed below. Some studies have reported no lasting effects of either right or left unilateral hippocampal lesions in spatial learning (Li et al., 1999, Li et al., 2012), or equivalent impairments after left and right functional inactivation (Fenton and Bures, 1993), and there is evidence suggesting that the total volume of hippocampal tissue spared after a lesion is the critical determinant of spatial learning ability, not its location—i.e. left or right, bilateral or unilateral (de Hoz et al., 2005). However, there is also some support for the idea that the left hippocampus plays a greater role in spatial learning processes—such as the acquisition (but not retrieval) of water maze place memory in young adult rats (Klur et al., 2009), and in radial maze performance, but only in aged rats (Poe et al., 2000). Functional asymmetries in the circuitry or recruitment of the left and right hippocampus might underlie some of these differences, but the existence of lateralized differences in synaptic plasticity is also a possibility.

According to the ‘classical’ view of the intrinsic hippocampal circuitry, connectivity follows a trisynaptic loop, predominantly in the transverse direction, perpendicular to the septotemporal axis (Andersen et al., 1971). However, in the rodent hippocampus at least, CA3-CA1 projections are extensively collateralized and longitudinal fibers can travel for large distances from their cells of origin (Amaral and Witter, 1989). As well as long-range intra-hippocampal Schaffer collaterals, a population of inter-hemispheric fibers crosses the midline via the ventral hippocampal commissure and makes contralateral synapses in CA1. Many CA3 neurons give rise to both ipsilateral Schaffer collateral and contralateral commissural projections (Swanson et al., 1981, Laurberg and Sørensen, 1981, Li et al., 1994). The extensive nature of the latter projection is evident in the large evoked potentials that can be recorded in CA1 following electrical stimulation of contralateral CA3 (Bliss et al., 1983, Stäubli and Lynch, 1987, Stäubli and Scafidi, 1999). Commissural projections to the stratum oriens of CA1 are typically denser than those evident in the stratum radiatum—the opposite of the pattern observed in the ipsilateral projection. But afferents originating in the intrahilar region of CA3, the region stimulated in this study, predominantly target the stratum radiatum both contralaterally and ipsilaterally (Laurberg, 1979). However, recent work has uncovered some intriguing asymmetries in the CA1 synapses of left versus right CA3 neurons: in the apical dendrites of mouse CA1, afferents originating from the left hemisphere preferentially innervate smaller spines with a high density of NMDA GluN2B subunits—necessary for the induction of persistent LTP *in vivo* (Ballesteros et al., 2016)—whereas projections originating on the right tend to target larger, mushroom-shaped spines, with higher densities of AMPA GluA1 receptor subunits (Kawakami et al., 2003, Wu et al., 2005, Shinohara et al., 2008, Shinohara and Hirase, 2009). This is true in both ipsilateral Schaffer collateral projections, and in contralateral commissural fibers.

Consistent with the anatomical picture, electrophysiological studies in mice have revealed a functional asymmetry, with projections from the left CA3 exhibiting a greater capacity for long-term potentiation (LTP)—a potential analog of the synaptic enhancement that occurs during learning (Martin and Morris, 2001, Martin and Morris, 2002, Morris et al., 2013)—than projections originating in right CA3. This was true of both LTP induced by a spike-timing-dependent pairing protocol (Kohl et al., 2011), or by a conventional high-frequency tetanus (Shipton et al., 2014). Selective activation of presynaptic afferents originating from one hemisphere was achieved using viral transfection of left versus right CA3 neurons with channelrhodopsin-2 followed by selective *ex vivo* optogenetic activation of the left and right projections. Electrical test stimulation, in contrast, evokes a mixed population of Schaffer collateral and commissural afferents in slices from both left and right hippocampus, and left–right asymmetries in LTP are therefore not observed. A follow-up study revealed a functional dissociation between left and right CA3 in hippocampus-dependent memory, with only left CA3 playing a role in an associative long-term place memory task (Shipton et al., 2014; for review, see El-Gaby et al., 2015).

Despite the growing evidence for asymmetries in the mouse CA3-CA1 projection, there is, to our knowledge, no corresponding evidence regarding the rat hippocampus. In the past, we have used the existence of ipsilateral and contralateral CA3-CA1 projections to provide treatment versus control pathways in the intact animal. By alternating stimulation of left and right CA3 *in vivo*, it is possible to record independent Schaffer collateral and commissural projections to CA1 (cf. Stäubli and Lynch, 1987). Recording and stimulating bilaterally yields a total of 4 pathways. Using this set-up, left versus right selectivity is achieved by the separate placement of left and right recording electrodes, rather than selective optogenetic stimulation. To address the question of CA3-CA1 asymmetry in rats, we now present a re-analysis of our existing *in vivo* CA1 data (Shires et al., 2012) to determine whether the properties of rat CA3-CA1 projections are influenced by the hemisphere in which they originate.

## Experimental procedures

### Animals

All procedures involving animals were conducted in accordance with the UK Animals (Scientific Procedures) Act (1986), and subject to the European Communities Council Directive of 24 November 1986 (86/609/EEC) and local ethical review. Prior to the experiment, adult male Lister-hooded rats (250–500 g), obtained from Charles River, UK, were pair-housed and given *ad libitum* access to food (standard rat chow) and water. They were provided with wood shavings and paper nesting material as bedding, given cardboard tubes for gnawing and refuge, and maintained on a 12-h light/12-h dark cycle. Cage dimensions were 32 × 50 cm.

### Surgery

At the start of an experiment, rats were anesthetized with urethane (ethyl carbamate; 1.5 g/kg; 0.3 mg/ml, IP), injected with carprofen (Rimadyl small animal solution, 4 mg/kg; SC), and placed in a stereotaxic frame with the skull horizontal. Body temperature was monitored by a rectal probe and maintained at 36.2 °C using an isothermic heating blanket. Depth of anesthesia was assessed throughout the experiment, and urethane top-ups of 0.2 ml were administered as required. Breathing rate was monitored continuously using a light-dependent resistor to detect thoracic movements, and analyzed online using in-house software. If breathing fell below 70 breaths per min, rats received an injection of atropine (0.4 mg/kg; SC) or doxapram (5 mg/kg; IP). Subcutaneous injections of a glucose/saline mixture were administered every 3 h to maintain hydration (1.5 ml of 0.9% saline + 0.5 ml of 5% glucose).

### Electrophysiological recording

**Fig. 1:**
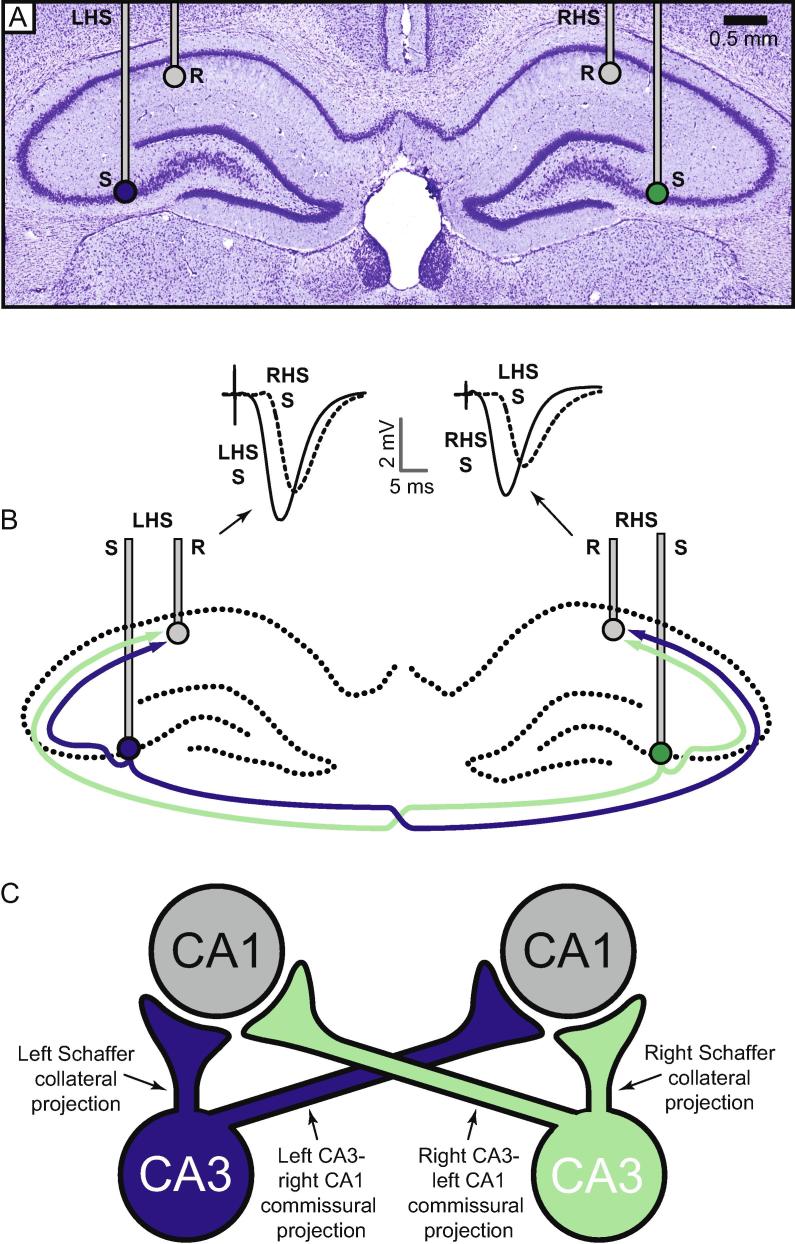
(A) Photomicrograph at the approximate AP location of the stimulation (S) and recording (R) electrodes. (B) Schematic illustration of the hippocampus, showing recording electrode locations (R), stimulating electrode locations (S), and pathways stimulated. In reality, CA3 stimulating electrodes were placed 0.3 mm anterior to CA1 recording electrodes, as well as 0.5 mm laterally. The path of the crossed commissural projections is not intended to be anatomically faithful; these projections travel via the ventral hippocampal commissure, and cannot be represented in a coronal section. Examples of fEPSPs recorded in the left hippocampus in response to ipsilateral left CA3-CA1 Schaffer collateral stimulation (LHS S) and contralateral right CA3-CA1 commissural stimulation (RHS S) are shown. (C) Cartoon of the four pathways analyzed in this study: left (blue) and right (green) uncrossed CA3-CA1 Schaffer collateral projections, left-to-right CA3-CA1 commissural projections (blue), and right-to-left CA3-CA1 commissural projections (green). (For interpretation of the references to color in this figure legend, the reader is referred to the web version of this article.)

PTFE-insulated monopolar platinum/iridium recording electrodes (external diameter = 0.103 mm) were lowered bilaterally into the stratum radiatum of area CA1 (3.8 mm posterior and 2.5 mm lateral to bregma; depth approximately −2.5 mm from the dura). Bipolar stimulating electrodes comprising two twisted wires identical in composition to the recording electrodes were lowered bilaterally into CA3 (3.5 mm posterior and 3.0 mm lateral to bregma; depth approximately −3.0 mm from dura) in order to activate independent populations of synaptic contacts made by ipsilateral Schaffer collateral and contralateral commissural projections converging on the same neuronal populations sampled by each of the recording electrodes. Fig. 1A shows a photomicrograph taken at the approximate antero-posterior level of the stimulating and recording electrodes; target electrode locations are indicated, and CA3-CA1 projections activated are shown in Fig. 1B. See Shires et al. (2012), Fig. 7, for a map of each individual stimulation and recording site. A simplified, schematic illustration of the electrode locations and pathways activated is shown in Fig. 1C; CA3–CA3 projections are omitted.

Evoked field potentials were amplified and filtered (high pass = 1 Hz; low pass = 5 kHz) using a differential AC amplifier (Model 1700, A-M Systems, Sequim, WA, USA), and sampled at 20 kHz using a data acquisition card (PCIe-6321; National Instruments, Austin, TX, USA) mounted in a PC running custom-written LabView software for the control of electrical stimulation and the time-locked recording of evoked fEPSPs (Evoked Potential Sampler, Patrick Spooner, University of Edinburgh). This program calculates a range of fEPSP measures, such as amplitude and slope (measured by linear regression between two fixed time-points). Stimulation was delivered via a NeuroLog system and stimulus-isolator units (Digitimer, Welwyn Garden City, UK), and consisted, during the main experiment, of biphasic constant-current pulses delivered alternately to left and right CA3. At the start of each experiment, electrodes were lowered into the hippocampus, and depths were adjusted to maximize the amplitude of the negative-going dendritic fEPSPs elicited in CA1 by stimulation of CA3. Stimulation intensity was adjusted to elicit a contralateral fEPSP of ∼3 mV in amplitude (200–500 μA), during an initial input–output curve. This yielded fEPSPs with slope values that were typically around 50% of maximum in both ipsilateral and contralateral pathways. Representative fEPSPs are shown in Fig. 1B.

The independence of commissural and Schaffer collateral pathways was confirmed at the start of the experiment, in each animal, for each hemisphere, by the delivery of pairs of biphasic stimulation pulses (50 μs per phase) to the commissural projection followed by the Schaffer collateral projection at an interval of 50 ms (six pairs; 10-s inter-pair interval), followed by single test pulses delivered to the Schaffer collateral projections only (6 pulses). Paired-pulse facilitation (PPF) was absent in all cases (see Shires et al., 2012, Figs. 2 and 4). After electrode placement, and the check for the absence of PPF, baseline recording began; single biphasic test pulses (50-μs pulse-width per phase; 0.1 ms total pulse-width) were delivered alternately to each stimulating electrode at 2-min intervals. Left and right CA3 stimulation sites were assigned as tetanized or control pathways in a quasi-random fashion. After a baseline period typically lasting several hours, and once stable fEPSPs had been observed for at least 1 h, unilateral tetanic stimulation was delivered. Total pulse-width was increased to 0.2 ms during a high-frequency tetanus. Rats received either a strong tetanus comprising three trains of 50 pulses at 250 Hz, with a 5-min inter-train interval, or a weak tetanus comprising 1 train of 50 pulses at 100 Hz. Data obtained from a specific recording electrode were discounted if the fEPSP slope elicited by ipsilateral or contralateral CA3 stimulation fell to 60% of the baseline value or below within 4–5 h of tetanization (or the corresponding time point for non-tetanized pathways). In most animals, therefore, both uncrossed and crossed CA3-CA1 projections were sampled in both hemispheres; however, data from only one hippocampus were available in some rats. In all cases, fEPSP slope data were normalized to the mean of the 1-h baseline period (assigned a value of 100%), and group means were calculated.

In an additional group of rats (*n* = 3), we implanted bilateral CA3 stimulating electrodes and a unilateral CA1 recording electrode as described above, before assessing both intra-pathway and cross-pathway PPF over a range of stimulation pulse-widths. The recording electrode was located in left CA1 in 2 rats, and right CA1 in one rat. As the stimulation pulse-width was doubled during tetanization, we wanted to ensure that PPF that was absent at a test-pulse duration of 0.1 ms did not appear at the tetanus pulse-width of 0.2 ms. As before, pairs of biphasic stimulation pulses were delivered to the commissural projection followed by the ipsilateral Schaffer collateral projection at an interval of 50 ms; the delivery of paired stimulation was always followed by the delivery of a single pulse to the ipsilateral pathway to provide baseline values for the calculation of cross-pathway PPF (six pairs and six single ipsilateral pulses; 10-s inter-pulse/pair interval). This was carried out using pulse-widths (both phases) of 0.1 ms, 0.2 ms, and 0.4 ms. PPF was calculated as: [(mean ipsilateral fEPSP slope after contralateral stimulation/mean ipsilateral fEPSP slope without contralateral stimulation) * 100]. The procedure was then repeated using pairs of ipsilateral stimulation pulses at an interval of 50 ms delivered to the same Schaffer collateral pathway to assess intra-pathway PPF (six pairs of pulses; 10-s inter-pair interval). In this case, PPF was calculated as: [(mean slope of the second fEPSP/mean slope of the first fEPSP) * 100].

In the majority of experiments, the continuous broad-band local field potential (LFP) at each CA1 recording electrode was recorded by splitting the output of the amplifier and sampling the data via a separate capture card mounted in a second PC running custom-written LabView software for LFP capture and analysis developed by Patrick Spooner (University of Edinburgh). Automatic selection of a 2-s sample of the raw LFP trace was triggered to occur 30 s after the onset of a fEPSP (to prevent the contamination of LFP with evoked activity). Using the same software, each sample was temporally filtered using a Hanning window to prevent onset/offset artifacts, bandpass filtered between 0.5 and 200 Hz, notch filtered at 50 Hz to remove mains interference, and spectrally analyzed using the fast Fourier transform (FFT) algorithm. This resulted in a series of spectral plots for each recording session in which power spectral density was expressed as a function of frequency from 0-80 Hz, divided into 0.5-Hz bins. These data were then converted to log_10_ values, and mean values were calculated in specific frequency bands—low-amplitude irregular activity (LIA; 1–2 Hz) and theta (3–6 Hz). The current analysis focuses on the 60-min baseline period immediately prior to tetanic stimulation.

### Experimental groups

In Shires et al. (2012), we reported 3 sets of data in which strong tetanic stimulation was delivered. However, the experimental conditions varied slightly between these groups as follows. In one group, an intracerebroventricular infusion of artificial cerebrospinal fluid was delivered 15 min prior to strong tetanization of left or right CA3 (*n* = 10). In another group, the same unilateral strong tetanus was delivered without vehicle infusion (*n* = 11). In the third group (*n* = 13), weak tetanization of the projection from contralateral CA3 preceded strong ipsilateral tetanization—the latter pathway is the sole focus of the present analysis. Considering all of these 3 groups together, from a total of 34 rats, 26 strongly tetanized uncrossed Schaffer collateral pathways, and 29 strongly tetanized crossed commissural pathways met our inclusion criteria (see above). In 21 of these cases, both ipsilateral and bilateral projections met criteria for inclusion (i.e. all 4 possible pathways were sampled). Comparing data from these 3 groups, there was no significant difference in the magnitude of LTP recorded 4–5 h after strong tetanization [*F*(2,49) = 0.90; *p* = 0.35]. This analysis confirms that the variations in experimental conditions had no impact on the amount of LTP observed, and the groups were therefore pooled for the analysis of left–right asymmetries.

A single homogeneous group of rats received a weak unilateral CA3 tetanus. From a total of 13 animals, 11 tetanized uncrossed Schaffer collateral pathways and 12 tetanized crossed commissural pathways met our criteria for inclusion. In 10 of these cases, both ipsilateral and bilateral projections met our criteria.

### Histology

At the end of all experiments, marking lesions were made by the delivery of biphasic 1-mA constant-current pulses (1 s per phase) to both stimulating and recording electrodes. Rats were killed by cervical dislocation and brains were removed and stored in 10% formalin. Thirty-micrometer coronal sections through the hippocampus were then cut using a cryostat: 1 in 3 sections was mounted on a slide and stained with cresyl violet. After examination under a light microscope, stimulation sites were marked on the appropriate coronal section taken from the Paxinos and Watson (2005) atlas. All electrodes were correctly positioned in CA3 and CA1 (see Shires et al., 2012).

### Data analysis and statistics

Numerical data were analyzed using Microsoft Excel and SPSS. Graphs were prepared using Excel, Origin, and Adobe Illustrator. Data are displayed as mean ± 1 standard error of the mean (SEM) throughout. Where effect sizes are reported, Cohen’s *d* was calculated by dividing the mean difference between the two groups to be compared by the pooled standard deviation.

## Results

**Fig. 2:**
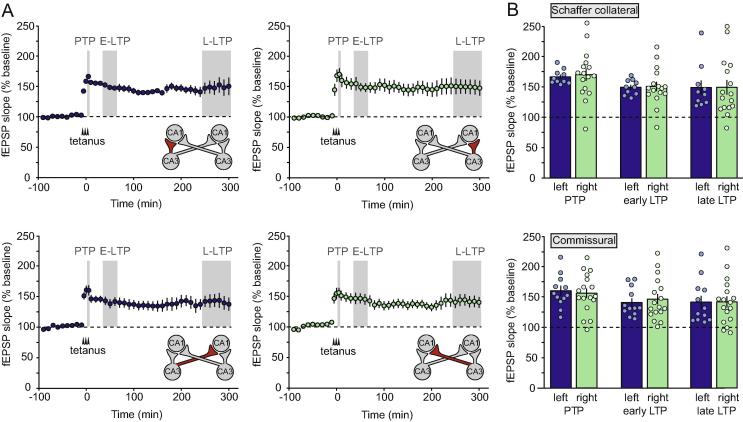
LTP induced by a strong tetanus. (A) Time course of LTP (relative to a tetanus at time ‘zero’) induced by a strong tetanus to left CA3 (left-hand panels) versus right CA3 (right-hand panels). Uncrossed Schaffer collateral pathways [top panels; *n* = 10 (left) and *n* = 16 (right)] and crossed commissural projections [bottom panels; *n* = 12 (left) and *n* = 17 (right)] are plotted separately. The illustration accompanying each graph shows the relevant pathway in red. The shaded areas indicate PTP (0-5 min post-tetanus); early LTP (E-LTP; 30-60 min post-tetanus) and late LTP (L-LTP; 4-5 h post-tetanus). (B) Percentage PTP, early LTP and late LTP in all 4 pathways. (For interpretation of the references to color in this figure legend, the reader is referred to the web version of this article.)

Strong tetanization of CA3 resulted in similar LTP in all 4 pathways examined: left CA3-CA1 uncrossed Schaffer collateral (*n* = 10), right CA3-CA1 uncrossed Schaffer collateral (*n* = 16); left CA3-right CA1 crossed commissural projection (*n* = 12), and right CA3-left CA1 crossed commissural projection (*n* = 17) (Fig. 2A). Separate two-way ANOVAs of the effects of tetanized hemisphere (left versus right) and pathway (uncrossed Schaffer collateral versus crossed commissural) were carried out for post-tetanic potentiation (PTP; 0–5 min post-tetanus), early LTP (30–60 min post-tetanus), and late LTP (4–5 h post-tetanus); see Fig. 2B. No differences between left versus right tetanization were observed [PTP: *F*(1,51) = 0.00; *p* = 0.99; effect size (Cohen’s *d*) = 0.00; early LTP: *F*(1,51) = 0.17; *p* = 0.68; effect size (Cohen’s *d*) = 0.13; late LTP: *F*(1,51) = 0.00; *p* = 0.97; effect size (Cohen’s *d*) = 0.02]. There was also no significant difference between crossed and uncrossed pathways [PTP: *F*(1,51) = 1.34; *p* = 0.25; effect size (Cohen’s *d*) = 0.35; early LTP: *F*(1,51) = 0.67; *p* = 0.42; effect size (Cohen’s *d*) = 0.22; late LTP: *F*(1,51) = 0.44; *p* = 0.51; effect size (Cohen’s *d*) = 0.19]. Potentiation was, however, numerically slightly lower in the crossed commissural projections.

**Fig. 3:**
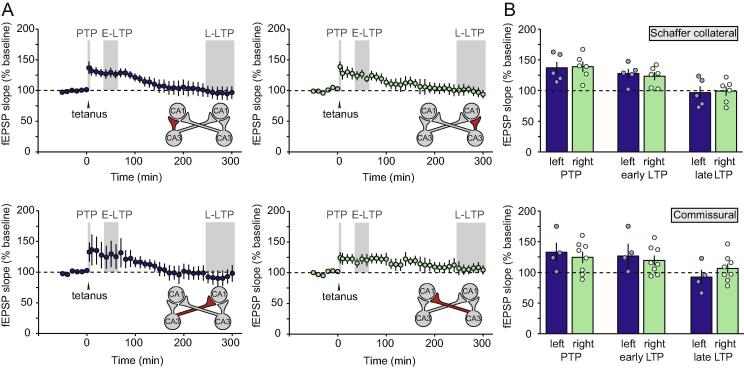
LTP induced by a weak tetanus. (A) Time course of LTP (relative to a tetanus at time ‘zero’) induced by a weak tetanus to left CA3 (left-hand panels) versus right CA3 (right-hand panels). Uncrossed Schaffer collateral pathways [top panels; *n* = 5 (left); *n* = 6 (right)] and crossed commissural projections [bottom panels; *n* = 4 (left); *n* = 8 (right)] are plotted separately. The illustration accompanying each graph shows the relevant pathway in red. The shaded areas indicate PTP (0-5 min post-tetanus); early LTP (E-LTP; 30-60 min post-tetanus) and late LTP (L-LTP; 4-5 h post-tetanus).(B) Percentage PTP, early LTP and late LTP (4–5 h post tetanus) in all 4 pathways. (For interpretation of the references to color in this figure legend, the reader is referred to the web version of this article.)

Weak tetanization of CA3 led to decaying early LTP that reached baseline within approximately 3 h in all 4 pathways: left CA3-CA1 uncrossed Schaffer collateral (*n* = 5), right CA3-CA1 uncrossed Schaffer collateral (*n* = 6); left CA3-right CA1 crossed commissural projection (*n* = 4), and right CA3-left CA1 crossed commissural projection (*n* = 8) (Fig. 3A). ANOVAs carried out as described above revealed no significant difference in potentiation after left versus right tetanization [PTP: *F*(1,19) = 0.10; *p* = 0.76; effect size (Cohen’s *d*) = 0.19; early LTP: *F*(1,19) = 0.17; *p* = 0.90; effect size (Cohen’s *d*) = 0.25; late LTP: *F*(1,19) = 0.79; *p* = 0.39; effect size (Cohen’s *d*) = 0.41], and no significant differences between crossed and uncrossed pathways [PTP: *F*(1,19) = 0.74; *p* = 0.40; effect size (Cohen’s *d*) = 0.45; early LTP: *F*(1,19) = 0.54; *p* = 0.82; effect size (Cohen’s *d*) = 0.15; late LTP: *F*(1,19) = 0.03; *p* = 0.86; effect size (Cohen’s *d*) = 0.19]; mean data are shown in Fig. 3B.

In the 1-h period before tetanization, there were no overall differences in mean fEPSP slope values elicited by stimulation of left versus right CA3 [ipsilateral Schaffer collateral: left CA3 = −1.09 ± 0.13 mV/ms; right CA3 = −1.18 ± 0.10 mV/ms; contralateral commissural: left CA3 = −0.76 ± 0.06 mV/ms; right CA3 = −0.72 ± 0.09 mV/ms; overall left versus right comparison: *F*(1,74) = 0.07; *p* = 0.79], but a highly significant difference between the fEPSP slopes elicited by contralateral (crossed) versus ipsilateral (uncrossed) CA3 stimulation [*F*(1,74) = 15.3; *p* < 0.0005]. Although the commissural CA3-CA1 projection is very extensive, fEPSPs elicited by stimulation of this pathway are typically smaller and exhibit longer latencies than those evoked by ipsilateral Schaffer collateral stimulation; examples are shown in Fig. 1B. There was no significant difference in the mean left and right stimulation intensities used to elicit these fEPSPs [ipsilateral Schaffer collateral: left CA3 = 433.3 ± 24.2 µA; right CA3 = 436.4 ± 21.6 µA; contralateral commissural: left CA3 = 435 ± 22.8 µA; right CA3 = 434.0 ± 21.2 µA; *F*(1,74) = 0.001; *p* = 0.98].

**Fig. 4:**
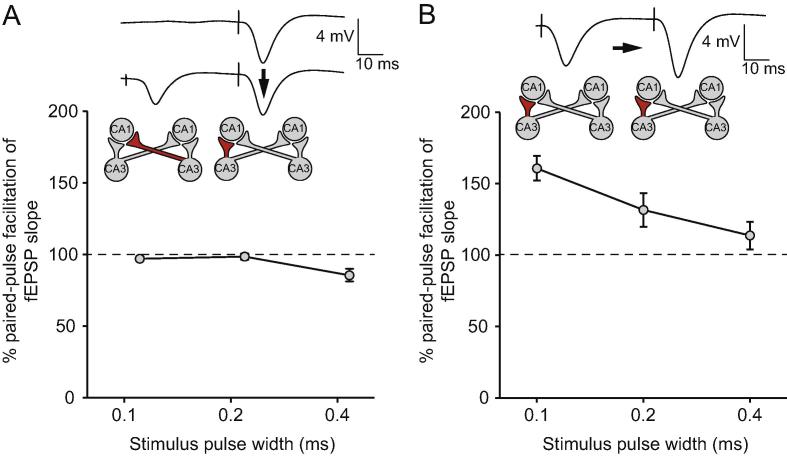
(A) Crossed-pathway stimulation of contralateral CA3 followed by ipsilateral CA3 at an interval of 50 ms did not result in PPF of fEPSPs recorded in ipsilateral CA1. An example of an ipsilateral CA3-CA1 fEPSP is shown without prior stimulation of the contralateral pathway (upper trace) and with prior contralateral stimulation (lower trace). The pulse-width was 0.2 ms in these examples, and the black arrow indicates the relevant comparison for the calculation of PPF. The schematic figures below the traces indicate that, in this example, the crossed pathway originated in right CA3, whereas the ipsilateral pathway originated in left CA3. The graph below shows percentage PPF as a function of pulse-width (B) Paired pulses delivered to the ipsilateral Schaffer collateral pathway at an interval of 50 ms caused robust PPF. An example is shown at a pulse-width of 0.2 ms; the black arrow highlights the key comparison between the slope of the first and second fEPSPs. The schematic figures below the trace indicates that, in this example, paired stimulation was delivered to the left CA3-CA1 projection. The graph below shows percentage PPF as a function of pulse-width. PPF declined with increasing pulse-width, but robust PPF was observed in all rats at pulse-widths of 0.1 and 0.2 ms.

Fig. 4A shows the effects of ‘cross-pathway’ paired-pulse stimulation of the contralateral CA3-CA1 projection followed by the ipsilateral Schaffer collateral projection at an interval of 50 ms. Owing to the low sample size and unilateral placement of recording electrodes in this experiment (left hippocampus: *n* = 2; right hippocampus: *n* = 1; bilateral stimulation in all cases; see Experimental Procedures), we focus solely on the independence of ipsilateral and contralateral projections as assessed by PPF, rather than an analysis of left versus right hippocampal responses. Cross-pathway stimulation did not result in PPF at test-pulse widths of 0.1 ms (the value used during low-frequency test stimulation during the main experiment) or 0.2 ms (the value used during tetanization). Group statistics were not conducted owing to the low number of animals, but individual comparisons in each animal of the slope of the second, ipsilateral, fEPSP in the presence or absence of prior contralateral stimulation did not reveal significant differences in any rat at either test intensity [*p* > 0.4 in all cases; paired sample *t*-tests]. At a pulse-width of 0.4 ms, higher than that used at any point in the current study, a significant paired-pulse depression was observed in all animals [*p* < 0.005 in all cases; paired sample *t*-tests with Bonferroni correction for multiple comparisons]. Conventional paired-pulse stimulation of the ipsilateral CA3-CA1 pathway (Fig. 4B) resulted in robust PPF at 0.1 ms [*p* < 0.05 in all cases; paired sample *t*-tests with Bonferroni correction for multiple comparisons] and 0.2 ms pulse-widths [*p* < 0.002 in all cases; paired sample *t*-tests with Bonferroni correction for multiple comparisons]. PPF was weaker at a 0.4-ms pulse-width, with only 2/3 rats exhibiting a significant increase [*p* < 0.02 in both cases; paired sample *t*-tests with Bonferroni correction for multiple comparisons]. Simulation currents were selected as described for the main experiment, and comparable values were chosen (mean ipsilateral stimulation current = 383.3 ± 72.6 µA; mean contralateral stimulation current = 400.0 ± 57.7 µA).

**Fig. 5:**
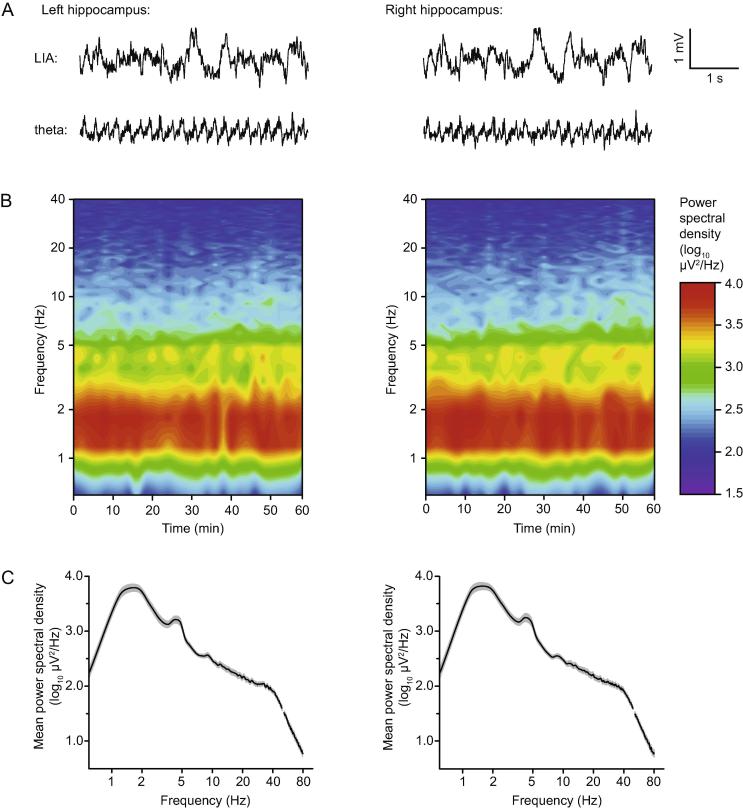
LFP activity in the left and right hippocampus of the same animals. (A) Samples of raw LFP trace illustrating corresponding episodes of LIA and theta activity in the left and right hippocampus of the same rat. (B) Mean time–frequency spectrum over the 60-min baseline period before the first tetanus in the left and right hippocampus (left and right panels respectively; *n* = 20). Each plot comprises 30 2-s samples of LFP recorded at 2-min intervals, and averaged across all 20 animals. (C) Mean power spectral density in log units [log_10_(µV^2^/Hz)] averaged over the full 1-h baseline period, and plotted as a function of frequency (log scale) for both left and right hippocampus (left and right panels respectively; *n* = 20). The shaded area represents ± 1 SEM.

Of the 31 rats in which both crossed and uncrossed CA3-CA1 inputs met our criteria for inclusion on both sides of the brain, we made continuous LFP recordings during the 1-h period before tetanization in 20 animals, allowing a direct comparison between LFP activity in the left and right hippocampus. The LFP was dominated by large-amplitude irregular activity (LIA; cf. Vanderwolf, 1969), with intermittent episodes of type II theta (cf. Kramis et al., 1975). Corresponding 5-s samples of each type of activity, taken from the left and right hippocampus of the same animal at different times, are shown in Fig. 5A; left and right hippocampal activity is qualitatively very similar. Fig. 5B shows mean time–frequency plots of 0–40 Hz activity over the 1-h period before the delivery of the first tetanus in the left and right hippocampi (left- and right-hand panels respectively). Mean power spectra averaged over the entire 1-h baseline period are shown in Fig. 5C. Note the broad low-frequency LIA peak, and the smaller peak between 4 and 5 Hz corresponding to intermittent theta activity. There were no significant left–right differences in mean spectral power [values expressed as log_10_(µV^2^/Hz) in all cases] over the LIA (1–2 Hz) and theta (3–6 Hz) ranges [LIA: left = 3.72 ± 0.07; right = 3.76 ± 0.07; *t*(19) = 1.91; *p* = 0.07; theta: left = 3.05 ± 0.05; right = 3.05 ± 0.06; *t*(19) = 0.06; *p* = 0.95; paired-sample *t*-tests].

## Discussion

Previous work in *ex vivo* mouse hippocampal slices has revealed that the synapses formed by presynaptic CA3-CA1 afferents originating in the left hemisphere are substantially more plastic than those originating on the right (Kohl et al., 2011, Shipton et al., 2014). These studies utilized optogenetic stimulation to sample the distinct populations of fibers originating from each hemisphere. However, after analyzing a previous dataset in which bilateral CA3 stimulation and CA1 recording were carried out in intact rats (see Shires et al. 2012), we did not find any left–right asymmetries in the capacity for LTP. Both left and right uncrossed Schaffer collateral projections showed equivalent potentiation after both strong tetanization that induces late LTP, and weak tetanization that results in a decaying early LTP. The same was true of the long-range crossed CA3-CA1 commissural projections originating in left and right CA3.

We cannot rule out the possibility that differences in the physiology and biophysics of optogenetic versus electrical stimulation might explain these differences in the laterality of LTP. The key drawback of electrical stimulation is its lack of selectivity in activating specific neuronal populations. For example, electrical stimulation causes the firing of local circuit neurons, as well as non-glutamatergic neurons and fibers of passage. These may include the axons of dopaminergic cells that have been implicated in the induction of persistent LTP (e.g. Sajikumar and Frey, 2004). However, our placement of stimulating electrodes in CA3, rather than in CA1 adjacent to the recording electrode, makes the co-activation of glutamatergic and dopaminergic terminals a less likely scenario in the current experiments.

Nonetheless, our conclusion that left and right Schaffer collateral projections can both support robust LTP depends on the assumption that the ipsilateral pathways are not appreciably contaminated by the recruitment of commissural projections from contralateral CA3. Our observation of pathway-specific LTP induction supports this view; contralateral control pathways exhibited no short- or long-term changes after an ipsilateral CA3 tetanus. However, ipsilateral placement of stimulating and recording electrodes in CA1, rather than CA3, does indeed result in the sampling of a mixed population of Schaffer collateral and commissural afferents running in the vicinity of the stimulating electrode, both *in vitro* and *in vivo*. In the intact rat, paired-pulse facilitation is evident when ipsilateral CA1 and contralateral CA3 are stimulated alternately at a 50-ms interval, indicating that the pathways are not fully independent under these circumstances, and the ipsilateral stimulator recruits a substantial commissural component; however, when stimulating electrodes are placed bilaterally in CA3 as in the current study, no paired-pulse facilitation is observed, implying that the pathways are non-overlapping in this configuration (Shires et al., 2012). A potential issue, however, is that we doubled the stimulation pulse-width from 0.1 to 0.2 ms during a tetanus in this experiment. We have found that doubling the pulse-width during tetanic stimulation is more effective for inducing robust and persistent LTP than, for example, doubling the stimulation current, perhaps because of the potentially damaging consequences of high stimulation currents (cf. Martin et al., 2013). However, this change will have increased the number of afferents recruited during tetanization, relative to those sampled during baseline test stimulation, perhaps resulting in an increasing overlap of ipsilateral and contralateral pathways. To address this, we recorded responses to cross-pathway (contralateral followed by ipsilateral) paired-pulse stimulation of CA3 at a range of pulse-widths in an additional group of animals (see Fig. 4). PPF was not observed in any animal at pulse widths of 0.1 and 0.2 ms, the values used for baseline test-pulse and tetanus stimulation, respectively, in the main experiment. Robust PPF was obtained at both pulse-widths following intra-pathway stimulation of the ipsilateral CA3-CA1 projection. The use of a longer pulse-width—0.4 ms—resulted in reduced intra-pathway PPF in the ipsilateral pathway, and paired-pulse depression following cross-pathway stimulation, consistent with previous evidence that high-intensity paired stimulation can cause paired-pulse depression, even in the ipsilateral CA3-CA1 projection (Leung et al., 2008).

Further support for the view that recruitment of commissural CA3-CA1 afferents does not contribute substantially to the CA1 fEPSP elicited by ipsilateral CA3 stimulation comes from our observation that a knife-cut lesion of CA3, anterior to a CA3 stimulating electrode, and contralateral to a CA1 recording electrode, almost completely abolishes the commissural response in CA1, but has little effect on CA3-CA1 responses elicited by ipsilateral stimulation and recording in the opposite, non-lesioned hemisphere (Martin et al., 2008). For these reasons, we think it is unlikely that left and right ‘ipsilateral’ projections sampled in the current experiment include substantial numbers of commissural afferents.

An additional complication to the interpretation of our data comes from the existence of commissural CA3-CA3 projections, for simplicity not included in Fig. 1B. As well as orthodromic activation of CA3-CA1 projections, CA3 stimulation will also lead to antidromic activation of commissural CA3-CA3 fibers, at least some of which may give rise to CA3-CA1 collaterals (Bliss et al., 1983). However, stimulation of contralateral CA3 is far less effective in firing ipsilateral CA3 neurons compared to antidromic activation from contralateral CA1 (Buzsàki and Eidelberg, 1982). This is consistent with our observation that marked paired-pulse facilitation is evident at a CA1 recording site after cross-pathway stimulation of ipsilateral and contralateral CA1, but not after bilateral cross-pathway stimulation of CA3 (Shires et al., 2012). For these reasons, it is unlikely that the Schaffer collaterals of antidromically activated CA3-CA3 fibers contribute significantly to the populations sampled in this study.

A related issue concerns the lower spatial selectivity and likely greater recruitment of glutamatergic fibers of passage by electrical stimulation of CA3 in the intact animal, compared to the *ex vivo* optogenetic stimulation of afferents from defined neuronal populations. Consistent with this idea, electrical micro-stimulation of the ventral posteromedial thalamus leads to a more widespread and less selective activation of the barrel cortex relative to optogenetic stimulation, a result that is attributed to a greater activation of fibers of passage by electrical stimulation (Millard et al., 2015; see also Histed et al., 2009). For further discussion of some of the methodological considerations surrounding optogenetic stimulation, see Häusser (2014). In the hippocampus, a population of Schaffer collaterals travels for long distances along the septotemporal axis of the structure before terminating in CA1. Optogenetic stimulation will activate only the axons of CA3 pyramidal cells with cell bodies located at the site of viral transfection (both *in vivo*, and in *ex vivo* slices; cf. Kohl et al., 2011 & Shipton et al., 2014). Conversely, electrical stimulation is likely to engage a wider population of afferents originating at different septotemporal levels. But again, it is not clear how this difference can account for the absence of lateralized LTP in the present experiments, unless there are differences in the synaptic targets of afferents originating at different septotemporal levels.

Another factor that is relevant in determining the magnitude and properties of LTP is the presence or absence of anesthesia. Urethane inhibits the responses of AMPA and NMDA receptors (Hara and Harris, 2002), and causes a small reduction in hippocampal fEPSPs when administered *in vivo* (Gilbert and Mack, 1999). Consistent with these effects, stronger tetanisation parameters are required to induce LTP in urethane-anesthetized rats, compared to awake animals (Riedel et al., 1994). However, it is not clear how this alone can account for our failure to observe asymmetrical LTP since, as discussed above, cross-pathway paired-pulse interactions were absent using both the stimulation parameters employed for baseline and tetanic stimulation.

Our observation of robust LTP in afferents from both left and right CA3 is consistent with a previous study in which Schaffer collateral and commissural pathways were examined in urethane-anesthetized rats with unilateral kainic acid lesions of the CA3 field contralateral to a stimulating electrode in the remaining CA3 region (Bliss et al., 1983), removing the issue of achieving independent activation of afferents from the left or right hemisphere. The efficacy of the lesion was confirmed by the complete absence of CA3 fEPSPs on the lesioned side elicited by contralateral stimulation of the intact CA3 field. Despite this, equivalent LTP was still observed in both projections from the intact CA3 region. Although the effect of left versus right CA3 lesions was not explicitly addressed, an example is provided of a lesion made in the left hippocampus, suggesting that at least some experiments involved the recording of LTP in afferents originating solely from the right, a result that is not consistent with a marked lateralization of synaptic plasticity. Another example of left–right symmetry in rat hippocampal synaptic plasticity is provided by a recent study of a form of NMDA-receptor-dependent long-term depression (LTD) induced by low-frequency optogenetic stimulation of the CA3-CA1 projection *in vivo* (O’Riordan et al., 2018). This form of plasticity was not lateralized, with equivalent LTD observed in both left and right hippocampi. A lateralized facilitation of left hippocampal LTD was, however, observed in the presence of amyloid-ß.

In the course of many of our experiments, we made a continuous bilateral record of hippocampal LFP activity via the same electrode used to capture fEPSPs. Although asymmetries in LFP power were not specifically predicted under the present circumstances, we chose to examine this possibility. Activity was, in fact, very similar in both hemispheres, consisting of LIA activity interspersed with episodes of Type II theta, and we did not observe any differences in spectral power in either frequency band. Little gamma-frequency activity was evident in our study, but left–right differences in the power of hippocampal gamma oscillations have been observed under some circumstances. For example, increases in the power of gamma oscillations (and synapse density) have been reported in the right, relative to the left, hippocampus in rats subject to environmental enrichment, but not in isolation-reared animals (Shinohara et al., 2013). More recently, a lateralization of spontaneous gamma activity has been reported in rats anesthetized with a slightly lower dose of urethane than that used in our study (1.2 g/kg; Benito et al., 2016). Using multi-site electrodes, Benito and colleagues observed that right CA1 gamma oscillations originating from spontaneous Schaffer collateral activity were larger than those recorded in the left hippocampus, and gamma waves on the right tended to precede those recorded on the left.

It is, of course, possible that the absence of asymmetrical plasticity in this study reflects a genuine undocumented difference in the morphology and function of left and right CA3-CA1 synapses between rats and mice. Although these two species are sometimes regarded as interchangeable in research, they have undergone substantial evolutionary divergence since they last shared a common ancestor at least 12 million years ago (Springer et al., 2003). However, hippocampal morphology and connectivity are very similar in both species, with only minor exceptions such as a lack of interhemispheric entorhinal cortex – dentate gyrus projections in mice (van Groen et al., 2002). Likewise, the morphological and electrophysiological properties of CA1 pyramidal cells are very similar in both rodents (Routh et al., 2009). Nonetheless, substantial differences in the patterns of CA3 commissural connectivity have been reported between rats, guinea pigs, and rabbits (van Groen and Wyss, 1988), and the existence of physiological or anatomical differences between rats and mice remains a possible explanation for the apparent symmetry of LTP in the rat CA3-CA1 system.

## References

[b0005] Amaral D.G., Witter M.P. (1989). The three-dimensional organization of the hippocampus: a review of anatomical data. Neuroscience.

[b0010] Andersen P., Bliss T.V.P., Skrede K.K. (1971). Lamellar organization of hippocampal pathways. Exp Brain Res.

[b0015] Ballesteros J.J., Buschler A., Köhr G., Manahan-Vaughan D. (2016). Afferent input selects NMDA receptor subtype to determine the persistency of hippocampal LTP in freely behaving mice. Front Synaptic Neurosci.

[b0020] Bast T. (2011). The hippocampal learning-behavior translation and the functional significance of hippocampal dysfunction in schizophrenia. Curr Opin Neurobiol.

[b0025] Benito N., Martín-Vázquez G., Makarova J., Makarov V.A., Herreras O. (2016). The right hippocampus leads the bilateral integration of gamma-parsed lateralized information. Elife.

[b0030] Bliss T.V.P., Lancaster B., Wheal H.V. (1983). Long-term potentiation in commissural and Schaffer collateral CA1 cells: an in vivo study in the rat. J Physiol.

[b0040] Burgess N., Maguire E., O’Keefe J. (2002). The human hippocampus and episodic memory. Neuron.

[b0045] Buzsàki G., Eidelberg E. (1982). Convergence of associational and commissural pathways on CA1 pyramidal cells of the rat hippocampus. Brain Res.

[b0050] de Hoz L., Martin S.J. (2014). Double dissociation between the contributions of the septal and temporal hippocampus to spatial learning: the role of prior experience. Hippocampus.

[b0055] de Hoz L., Moser E.I., Morris R.G.M. (2005). Spatial learning with unilateral and bilateral hippocampal networks. Eur J Neurosci.

[b0060] El-Gaby M., Shipton O.A., Paulsen O. (2015). Synaptic plasticity and memory: new insights from hippocampal left-right asymmetries. Neuroscientist.

[b0065] Fenton A.A., Bures J. (1993). Place navigation in rats with unilateral tetrodotoxin inactivation of the dorsal hippocampus: place but not procedural learning can be lateralized to one hippocampus. Behav Neurosci.

[b0070] Gilbert M.E., Mack C.M. (1999). Field potential recordings in dentate gyrus of anesthetized rats: stability of baseline. Hippocampus.

[b0075] Hara K., Harris R.A. (2002). The anesthetic mechanism of urethane: the effects on neurotransmitter-gated ion channels. Anesth Analg.

[b0080] Häusser M. (2014). Optogenetics: the age of light. Nat Methods.

[b0085] Histed M.H., Bonin V., Reid C. (2009). Direct activation of sparse, distributed populations of cortical neurons by electrical microstimulation. Neuron.

[b0090] Kawakami R., Shinohara Y., Kato Y., Sugiyama H., Shigemoto R., Ito I. (2003). Asymmetrical allocation of NMDA receptor epsilon2 subunits in hippocampal circuitry. Science.

[b0095] Klur S., Muller C., Pereira de Vasconcelos A., Ballard T., Lopez J., Galani R., Certa U., Cassel J.C. (2009). Hippocampal-dependent spatial memory functions might be lateralized in rats: an approach combining gene expression profiling and reversible inactivation. Hippocampus.

[b0100] Kohl M.M., Shipton O.A., Deacon R.M., Rawlins J.N., Deisseroth K., Paulsen O. (2011). Hemisphere-specific optogenetic stimulation reveals left-right asymmetry of hippocampal plasticity. Nat Neurosci.

[b0105] Kramis R., Vanderwolf C.H., Bland B.H. (1975). Two types of hippocampal rhythmical slow activity in both the rabbit and the rat: relations to behavior and effects of atropine, diethyl ether, urethane, and pentobarbital. Exp Neurol.

[b0130] Laurberg S. (1979). Commissural and intrinsic connections of the rat hippocampus. J Comp Neurol.

[b0135] Laurberg S., Sørensen K.E. (1981). Associational and commissural collaterals of neurons in the hippocampal formation (hilus fasciae dentatae and subfield CA3). Brain Res.

[b0110] Leung L.S., Peloquin P., Canning K.J. (2008). Paired-pulse depression of excitatory postsynaptic current sinks in hippocampal CA1 in vivo. Hippocampus.

[b0140] Li X.G., Somogyi P., Ylinen A., Buzsáki G. (1994). The hippocampal CA3 network: an in vivo intracellular labeling study. J Comp Neurol.

[b0115] Li H., Matsumoto K., Watanabe H. (1999). Differential effects of unilateral and bilateral hippocampal lesions in rats on the performance of radial maze and odor-paired associate tasks. Brain Res Bull.

[b0120] Li H., Wu X., Bai Y., Huang Y., He W., Dong Z. (2012). Unilateral lesion of dorsal hippocampus in adult rats impairs contralateral long-term potentiation in vivo and spatial memory in the early postoperative phase. Behav Brain Res.

[b0125] Martin S.J., Bast T., Steffenach H.-A., Paterson A., Witter M., Morris R.G.M. (2008). Interruption of longitudinal transmission within hippocampal area CA3: implications for spatial memory. Soc Neurosci Abs.

[b0145] Martin S.J., Morris R.G.M. (2001). Cortical plasticity: It's all the range!. Curr Biol.

[b0150] Martin S.J., Morris R.G.M. (2002). New life in an old idea: the synaptic plasticity and memory hypothesis. Hippocampus.

[b0155] Martin S.J., Shires K.L., Spooner P.A. (2013). The relationship between tetanus intensity and the magnitude of hippocampal long-term potentiation in vivo. Neuroscience.

[b0160] Millard D.C., Whitmire C.J., Gollnick C.A., Rozell C.J., Stanley G.B. (2015). Electrical and optical activation of mesoscale neural circuits with implications for coding. J Neurosci.

[b0165] Morris R.G.M., Steele R.J., Bell J.E., Martin S.J. (2013). N-methyl-D-aspartate receptors, learning and memory: chronic intraventricular infusion of the NMDA receptor antagonist D-AP5 interacts directly with the neural mechanisms of spatial learning. Eur J Neurosci.

[b0170] O'Riordan K.J., Hu N.-W., Rowan M.J. (2018). Aß facilitates LTD at Schaffer collateral synapses preferentially in the left hippocampus. Cell Rep.

[b0175] Paxinos G., Watson C. (2005). The rat brain in stereotaxic coordinates.

[b0180] Poe G.R., Teed R.G., Insel N., White R., McNaughton B.L., Barnes C.A. (2000). Partial hippocampal inactivation: effects on spatial memory performance in aged and young rats. Behav Neurosci.

[b0185] Riedel G., Seidenbecher T., Reymann K.G. (1994). LTP in hippocampal CA1 of urethane-narcotized rats requires stronger tetanization parameters. Physiol Behav.

[b0190] Routh B.N., Johnston D., Harris K., Chitwood R.A. (2009). Anatomical and electrophysiological comparison of CA1 pyramidal neurons of the rat and mouse. J Neurophysiol.

[b0195] Sajikumar S., Frey J.U. (2004). Late-associativity, synaptic tagging, and the role of dopamine during LTP and LTD. Neurobiol Learn Mem.

[b0200] Shinohara Y., Hirase H. (2009). Size and receptor density of glutamatergic synapses: a viewpoint from left-right asymmetry of CA3-CA1 connections. Front Neuroanat.

[b0205] Shinohara Y., Hirase H., Watanabe M., Itakura M., Takahashi M., Shigemoto R. (2008). Left-right asymmetry of the hippocampal synapses with differential subunit allocation of glutamate receptors. Proc Natl Acad Sci USA.

[b0210] Shinohara Y., Hosoya A., Hirase H. (2013). Experience enhances gamma oscillations and interhemispheric asymmetry in the hippocampus. Nat Commun.

[b0215] Shipton O.A., El-Gaby M., Apergis-Schoute J., Deisseroth K., Bannerman D., Paulsen O., Kohl M.M. (2014). Left-right dissociation of hippocampal memory processes in mice. Proc Natl Acad Sci USA.

[b0220] Shires K.L., da Silva B.M., Hawthorne J.P., Morris R.G.M., Martin S.J. (2012). Synaptic tagging and capture in the living rat. Nat Commun.

[b0225] Springer M.S., Murphy W.J., Eizirik E., O’Brien S.J. (2003). Placental mammal diversification and the Cretaceous-Tertiary boundary. Proc Natl Acad Sci USA.

[b0035] Strange B.A., Witter M.P., Lein E.S., Moser E.I. (2014). Nat Rev Neurosci.

[b0230] Stäubli U., Lynch G. (1987). Stable hippocampal long-term potentiation elicited by 'theta' pattern stimulation. Brain Res.

[b0235] Stäubli U., Scafidi J. (1999). Time-dependent reversal of long-term potentiation in area CA1 of the freely moving rat induced by theta pulse stimulation. J Neurosci.

[b0240] Swanson L.W., Sawchenko P.E., Cowan W.M. (1981). Evidence for collateral projections by neurons in Ammon's horn, the dentate gyrus, and the subiculum: a multiple retrograde labeling study in the rat. J Neurosci.

[b0245] Vanderwolf C.H. (1969). Hippocampal electrical activity and voluntary movement in the rat. Electroencephalogr Clin Neurophysiol.

[b0250] van Groen T., Kadish I., Wyss J.M. (2002). Species differences in the projections from the entorhinal cortex to the hippocampus. Brain Res Bull.

[b0255] van Groen T., Wyss J.M. (1988). Species differences in hippocampal commissural connections: studies in rat, guinea pig, rabbit, and cat. J Comp Neurol.

[b0260] Wu Y., Kawakami R., Shinohara Y., Fukaya M., Sakimura K., Mishina M., Watanabe M., Ito I., Shigemoto R. (2005). Target-cell-specific left-right asymmetry of NMDA receptor content in Schaffer collateral synapses in epsilon1/NR2A knock-out mice. J Neurosci.

